# Supplementary material for: Analysis of Cross-Combinations of Feature Selection and Machine-Learning Classification Methods Based on [18F]F-FDG PET/CT Radiomic Features for Metabolic Response Prediction of Metastatic Breast Cancer Lesions
Source: Cancers (Basel). 2022 Jun 14;14(12):2922. doi: 10.3390/cancers14122922 (PMC9221062; doi:10.3390/cancers14122922)

Supplementary Material

Analysis of Cross-Combinations of Feature Selection and Machine-Learning Classification Methods Based on [18F]F-FDG PET/CT Radiomic Features for Metabolic Response Prediction of Metastatic Breast Cancer Lesions

Authors:

OBER VAN GOMEZ LOPEZ[1,2], obervang@ucm.es, orcid:0000-0003-1411-6197
JOAQUIN LOPEZ HERRAIZ[1], jlopezhe@ucm.es, orcid:0000-0001-7208-8863
JOSE MANUEL UDIAS MOINELO[1], jose@nuc2.fis.ucm.es, orcid:0000-0003-3714-764)
ALEXANDER HAUG[2], alexander.haug@meduniwien.ac.at, orcid:0000-0002-8308-6174
LASZLO PAPP[3], laszlo.papp@meduniwien.ac.at, orcid:0000-0002-9049-9989
DANIA CIONI[4], dania.cioni@med.unipi.it
EMANUELE NERI[4], emanuele.neri@med.unipi.it, orcid:0000-0001-7950-4559

(1) Nuclear Physics Group and IPARCOS, Faculty of Physical Sciences, University Complutense of Madrid, CEI Moncloa, 28040 Madrid, Spain.
(2) Academic Radiology and Master in Oncologic Imaging, Department of Translational Research, University of Pisa, Via Roma, 67, 56126 Pisa, Italy.
(3) Division of Nuclear Medicine, Department of Biomedical Imaging and Image-Guided Therapy, Medical University of Vienna, 1090 Vienna, Austria.
(4) Center for Medical Physics and Biomedical Engineering, Medical University of Vienna, 1090 Vienna, Austria.
(5) Italian Society of Medical and Interventional Radiology, SIRM Foundation, Via della Signora 2, 56122, Milan, Italy.

Authors for correspondence:

EMANUELE NERI
emanuele.neri@med.unipi.it
Master in Oncologic Imaging, Diagnostic and Interventional Radiology, Department of Translational Research, University of Pisa, Via Roma, 67, 56126 Pisa, Italy.

OBER VAN GOMEZ LOPEZ
obervang@ucm.es
Complutense University of Madrid, Faculty of Physical Sciences
Department of Structure of Matter, Thermal Physics and Electronics
Avda. Complutense, s/n

Short Title: Machine Learning prediction of metabolic response in metastatic breast cancer

Table S1. Summary of the radiomics features.

Category feature (number)	Name
**Intensity (13)**	SUVmax. SUVpeak. SUVmean. SUVstd. SUVvar. SUVenergy. AUC_CSH. Mean. Variance. Skewness. Kurtosis. Energy. Entropy-histogram
**Textural (88)**	
**GLCM**	Energy. Entropy. Difference entropy. Sum entropy. Variance1. Variance2. Sum variance. Max Possibility. Contrast. Dissimilarity. Homogeneity1. Homogeneity2. Correlation. DiffVar. Autocorrelation. Cluster prominence. Cluster shade. Cluster tendency. ICM1. ICM2. InVar. IDMN. IDN. Sum Average1. Sum Average2. Agreement
**GLRLM**	SRE. LRE. GLN. RLN. RP. LGRE. HGRE. SRLGE. SRHGE. LRLGE. LRHGE. GLV. RLV
**GLSZM**	SZE. LZE. GLN. ZSN. ZP. LGZE. HGZE. SZLGE. SZHGE. LZLGE. LZHGE. GLV. ZSV
**NGTDM**	Coarseness. Contrast. Busyness. Complexity. Strength
**GLGLM**	SGE. LGE. GLF. GaLN. GP. LGGE. HGGE. SGLGE. SGHGE. LGLGE. LGHGE. GrLV. GaLV
**NGLDM**	Entropy. Energy. SNE. LNE. NNU
**TS**	BWS. MasSpe
**TFC**	Coarseness. Mean Convergence. Variance
**TFCM**	Code Entropy. Code Similarity. Contrast. SAM. IDM. Homogeneity. Intensity. Entropy

Abbreviation:

Intensity
- SUV: standard uptake value
- AUC_CSH: Area under the curve of the cumulative SUV-volume histogram

GLCM (gray level co-occurrence matrix)
- DiffVar: difference variance

  Table A1. Summary of the radiomics features (***continuation***)
- ICM1: informational measure of correlation1
- ICM2: informational measure of correlation2
- InVar: inverse variance
- IDMN: inverse difference moment normalized
- IDN: inverse difference normalized

GLRLM (gray level run length matrix)
- SRE: short run emphasis
- LRE: long run emphasis
- GLN: gray-level non-uniformity
- RLN: run-length nonuniformity
- RP: run percentage
- LGRE: low gray-level run emphasis
- HGRE: high gray-level run emphasis
- SRLGE: short run low gray-level emphasis

- SRHGE: short run high gray-level emphasis
- LRHGE: long run high gray-level emphasis
- GLV: gray-level variance
- RLV: run-length variance

GLSZM (gray level size zone matrix)**:**
- SZE: small zone emphasis
- LZE: large zone emphasis
- GLN: gray-level non-uniformity
- ZSN: zone-size nonuniformity
- ZP: zone percentage
- LGZE: low gray-level zone emphasis
- HGZE: high gray-level zone emphasis
- SZLGE: small zone low gray-level emphasis
- SZHGE: small zone high gray-level emphasis
- LZLGE: large zone low gray-level emphasis
- LZHGE: large zone high gray-level emphasis
- GLV: gray-level variance
- ZSV: zone-size variance

NGTDN (neighborhood gray tone difference matrix)
GLGLM (gray-level run-length matrix)
- SGE: short gap emphasis
- LGE: long gaps emphasis
- GLF: gray level fluctuation
- GaLN: gap length nonuniformity
- GP: gap percentage
- LGGE: Low Gray-Level Gap Emphasis
- HGGE: High Gray-Level Gap Emphasis
- SGLGE: Short Gap Low Gray-Level Emphasis
- SGHGE: Short Gap High Gray-Level Emphasis
- LGLGE: Long Gap Low Gray-Level Emphasis
- LGHGE: Long Gap High Gray-Level Emphasis
- GrLV: Gray-Level Variance
- GaLV: Gap- Length Variance

NGLDM (neighboring gray level dependence matrix)
- SNE: Small number emphasis
- LNE: Large number emphasis
- NNU: number nonuniformity

TS (texture spectrum)
- BWS: black white symmetry
- MasSpe: Max spectrum

TFC (texture feature coding)
TFCM (texture feature coding method)
- SAM: Second angular moment
- IDM: inverse difference moment

Table S2. **Formulas and Description of some image features**

Morphological Features			
	**Parameter**	**Formula**	**Description**
**Shape and Size based features**	**Compactness**	$Compactness = \frac{V}{\sqrt{\pi}A^{\frac{3}{2}}}$ *Where V denote the volume and A denote the surface area of the volume of interest (VOI)*	Quantifies how close an object to the smoothest shape. the circle
	**Surface area**	$SA = \sum_{i=1}^{N} \frac{1}{2} \lvert a_i b_i \times a_i c_i \rvert$ *Where N is the total number triangle (coved surface area) and* $a.b.c$ *are edge vectors*	The surface area of the ROI
	**Convexity**	$Convexity = \frac{V}{V'}$ *Where V denote tumor volume and V′ denote convex hull volume*	Measures ratio of the ROI volume contained within the tumor to the calculated convex hull volume
	**Sphericity**	$Sphericity = \frac{36\pi \times (V^2)^{\frac{1}{3}}}{A}$ *Where A denote area and V denote tumor volume*	Measures of the roundness of the ROI
	**Maximum 3D diameter**	*See description in the next column*	Measures of the maximum 3D ROI diameter. It is measured as the largest pairwise Euclidean distance. between surface voxels of the ROI
	**Spherical disproportion**	$Spherical\ disproportion = \frac{A}{4\pi R^2}$ *Where R is the radius of a sphere with the same volume as the ROI*	The ratio of the surface area of the ROI to the surface area of a sphere with the same volume as the ROI
	**Surface to volume ratio (SVR)**	$SVR = \frac{A}{V}$ *Where A is area and V is volume*	Surface to volume ratio
**Physical based features**	**Volume**	$Volume = R * number\ of\ voxels$ *Where R denote the 3d image resolution*	Volume of tumor (ROI)

Textural Features			
	**Parameter**	**Formula**	**Description**
**First order features** (Histogram based features)	**Maximum**	$Max = max(X(i))$ *Where X denote the 3d image matrix*	Measures maximum intensity value of a histogram
	**Minimum**	$Min = min(X(i))$ *Where X denote the 3d image matrix*	Measures minimum intensity value of a histogram
	**Median**	$Median = \frac{X(i)}{2}$ *Where X denote the 3d image matrix*	Measures median intensity value of a histogram
	**Mean**	$Mean = \frac{1}{N}\sum_{i}^{N} X(i)$ *Where X denote the 3d image matrix with N voxel.*	Measures mean intensity value of a histogram
	**Variance**	$Variance = \frac{1}{N-1}\sum_{i=1}^{N} (X(i) - \bar{x})^2$	Measures squared distances of each value of a histogram from the mean
	Energy	$Energy = \sum_{i}^{N} X(i)^2$ *Where X denote the 3d image matrix with N voxel.*	Measures squared magnitude value of a histogram
	**Standard deviation**	$Std = \left(\frac{1}{N-1}\sum_{i=1}^{N} (X(i) - \bar{x})^2\right)^{1/2}$ *Where X denote the 3d image matrix with N voxel.*	Measures amount of variation of a histogram.
	**Skewness**	$Skewness = \frac{E(x-\mu)^3}{\sigma^3}$ *Where* μ *is the mean of* x. σ *is the standard deviation of* x. E *is the expectation operator.*	Measures asymmetry of a histogram.
	**Kurtosis**	$Kurtosis = \frac{E(x-\mu)^4}{\sigma^4}$ *Where* μ *is the mean of* x. σ *is the standard deviation of* x. E *is the expectation operator.*	Measures "peakedeness" of a histogram (flatness of histogram)
	**Root mean square (RMS)**	$RMS = \sqrt{\frac{1}{N}\sum_{n=1}^{N} \lvert X_n \rvert^2}$ *Where X denote the 3d image matrix with N voxel.*	Measures the square-root of the mean of the squares of the values of the histogram. This feature is another measure of the magnitude of a histogram
	**Inter quartile range**	$IQR = Q_3 - Q_1$ *Where* Q_3 *denote the 3rd quartile of histogram.* Q_1 *denote the 1st quartile of histogram*	Measures of variability. based on dividing a histogram into quartiles
	**Range**	$Range = range(X(i))$	Measures difference between the highest and lowest voxel values of a histogram

	**Entropy**	$$Entropy = -\sum_{i=1}^{N_l} P(i)\, log_2\, P(i)$$ *Where P denote the first order histogram with N_l discrete intensity levels.*	Measures irregularity of a histogram.
	**Uniformity**	$$Uniformity = \sum_{i=1}^{N_l} P(i)^2$$ *Where P denote the first order histogram with N_l discrete intensity levels.*	Measures uniformity of a histogram.
	**Percentile**	$$Percentile = \left(\frac{n^{th}\ percentile}{100}\right) X(i)$$	Measures intensity value at the 2.5th . 25th .50th .75th . and 97.5th percentile on histogram
**Second order textural features** (GLCM based features)	**Autocorrelation**	$$Autocorrelation = \sum_{i=1}^{N_g}\sum_{j=1}^{N_g} ij\boldsymbol{P}(i.j)$$	Measures of the magnitude of the fineness and coarseness of texture
	**Cluster tendency**	$$Cluster\ tendency = \sum_{i=1}^{N_g}\sum_{j=1}^{N_g}\left[i + j - \mu_x - \mu_y\right]^2 \boldsymbol{P}(i.j)$$	Measures of the homogeneity of GLCM
	**Maximum probability**	$$Maximum\ probability = max\{P(i.j)\}$$	Measures maximum value of GLCM matrix
	**Contrast**	$$Contrast = \sum_{i=1}^{N_g}\sum_{j=1}^{N_g} \lvert i - j\rvert^2 \boldsymbol{P}(i.j)$$	Measures of the local intensity variation of GLCM
	**Difference entropy**	$$Difference\ entropy = \sum_{i=0}^{N_g - 1} \boldsymbol{P}_{x-y}(i)\, log_2[P_{x-y}(i)]$$	Measures entropy of processed GLCM matrix Px-y
	**Dissimilarity**	$$Dissimilarity = \sum_{i=1}^{N_g}\sum_{j=1}^{N_g} \lvert i - j\rvert \boldsymbol{P}(i.j)$$	Measures differences of entries in GLCM
	**Energy**	$$Energy = \sum_{i=1}^{N_g}\sum_{j=1}^{N_g} [\boldsymbol{P}(i.j)]^2$$	Measures of the homogeneity of GLCM
	**Entropy**	$$Entropy = -\sum_{i=1}^{N_g}\sum_{j=1}^{N_g} \boldsymbol{P}(i.j)\, log_2[\boldsymbol{P}(i.j)]$$	Measures irregularity of GLCM
	**Homogeneity1**	$$Homogeneity1 = \sum_{i=1}^{N_g}\sum_{j=1}^{N_g} \frac{\boldsymbol{P}(i.j)}{1 + \lvert i - j\rvert}$$	Measures closeness of GLCM
	**Informational measure of correlation 1 (IMC1)**	$$IMC1 = \frac{HXY - HXY1}{max\{HX.HY\}}$$	Secondary measure of Homogeneity1
	**Sum entropy**	$$Sum\ entropy = -\sum_{i=2}^{2N_g} P_{x+y}(i)\, log_2\left[P_{x+y}(i)\right]$$	Sum of neighborhood intensity value differences

	**Variance**	$$Variance = \sum_{i=1}^{N_g}\sum_{j=1}^{N_g}(i-\mu_x)^2 P(i.j)$$	Measures dispersion of the parameter values around the mean of the combinations of reference and neighborhood pixels
	**Sum average**	$$Sum\ average\ = \sum_{i=2}^{2N_g}\left[iP_{x+y}(i)\right]$$	Measures the relationship between occurrences of pairs with lower and higher intensity values
	**Sum variance**	$$Sum\ variance = \sum_{i=2}^{2N_g}(i-SA)^2 P_{x+y}(i)$$	
	**Inverse variance**	$$inverse\ variance = \sum_{i=1}^{N_g}\sum_{j=1}^{N_g}\frac{P(i.j)}{\lvert i-j\rvert^2}.\ i \neq j$$	
	**Inverse Difference Moment Normalized (IDMN)**	$$IDMN = \sum_{i=1}^{N_g}\sum_{j=1}^{N_g}\frac{P(i.j)}{1+\left(\frac{\lvert i-j\rvert^2}{N^2}\right)}$$	Measures the local homogeneity of an image
	Where $\boldsymbol{P}(i.j)$ is the gray level co-occurrence matrix for ($\delta = 1. \alpha = 0$).<br>*N_g is the number of discrete intensity value in the image.*<br>*N is the number of voxels in the ROI.*<br>*μ is the mean of $\boldsymbol{P}(i.j)$.*<br>$p_x(i) = \sum_{j=1}^{N_g}\boldsymbol{P}(i.j)$ *are the marginal row probabilities.*<br>$p_y(i) = \sum_{i=1}^{N_g}\boldsymbol{P}(i.j)$ *are the marginal column probabilities.*<br>*μ_x is the expected value of marginal row probability.*<br>*μ_y is the expected value of marginal column probability.*<br>*σ_x is the standard deviation of p_x.*<br>*σ_y is the standard deviation of p_y.*<br>$p_{x+y}(k) = \sum_{i=1}^{N_g}\sum_{j=1}^{N_g}\boldsymbol{P}(i.j)\ .i+j = k.k = 2.3.\dots.2N_g.$<br>$p_{x-y}(k) = \sum_{i=1}^{N_g}\sum_{j=1}^{N_g}\boldsymbol{P}(i.j)\ .\lvert i-j\rvert = k.k = 0.1.\dots.N_g - 1.$<br>$HX = -\sum_{i=1}^{N_g}\boldsymbol{P}_x(i)\ log_2[p_x(i)]$ *is the entropy of $\boldsymbol{P}_x$.*<br>$HY = -\sum_{i=1}^{N_g}\boldsymbol{P}_y(i)\ log_2[p_y(i)]$ *is the entropy of $\boldsymbol{P}_y$.*<br>$HXY = -\sum_{i=1}^{N_g}\sum_{j=1}^{N_g}\boldsymbol{P}(i.j)\ log_2[\boldsymbol{P}(i.j)]$ *is the entropy of $\boldsymbol{P}(i.j)$*<br>$HXY1 = -\sum_{i=1}^{N_g}\sum_{j=1}^{N_g}\boldsymbol{P}(i.j)\ log(p_x(i)p_y(j)).$		
**Higher-order features**			
**Higher order features** (ISZ based features)	**Parameter**	Formula	Description
	**Size-zone variability**	$$\frac{1}{\Theta}\sum_{m=1}^{M}\left[\sum_{n=1}^{N}\boldsymbol{P}(m.n)\right]^2$$	Variability in the size
	**Intensity variability**	$$\frac{1}{\Theta}\sum_{n=1}^{N}\left[\sum_{m=1}^{M}\boldsymbol{P}(m.n)\right]^2$$	Variability in the intensity
	Where $\boldsymbol{P}(m.n)$ is the intensity size zone matrix<br>*Θ represents the number of homogeneous areas in the tumor.*<br>*M is the number of distinct intensity values.*<br>*N is the size of the homogeneous area in the matrix $\boldsymbol{P}(m.n)$*		

***Table S3.* Univariate Analysis**

No.	Variable	*p*-value (2-side)
	**Clinical variables**	
**1**	Age	0.472
**2**	T	0.005
**3**	N	0.039
**4**	Histology	0.531
**5**	ER	0.000
**6**	PR	0.000
**7**	Her2-new	0.003
**8**	Grading	0.024
**9**	Ki-67	0.005
	**Metabolic variables**	
**10**	SUV_peak	0.001
**11**	SUV_mean	0.018
**12**	SUV_min	0.262
**13**	SUV_max	0.017
**14**	SUV_StdDev	0.083
	**Image features**	
**15**	Mean_PET	0.042
**16**	Min_PET	0.838
**17**	Max_PET	0.041
**18**	Sum_PET	0.000
**19**	Std_Dev_PET	0.121
**20**	Variance_PET	0.256
**21**	Skewness_PET	0.668
**22**	Kurtosis_PET	0.057
**23**	Energy_PET	0.009
**24**	Energy_PET.1	0.985
**25**	Correlation_PET	0.000
**26**	Clusterprominence_PET	0.776
**27**	ICM1_PET	0.001
**28**	Variance_PET.1	0.911
**29**	C.MaxPossibility_PET	0.056
**30**	SGE_PET	0.000
**31**	GLF_PET	0.000
**32**	SGLGE_PET	0.809
**33**	LGHGE_PET	0.000
**34**	GrLV_PET	0.000
**35**	GaLV_PET	0.000
**36**	Energy_PET.2	0.000
**37**	GLV_PET	0.004
**38**	RLV_PET	0.000
**39**	ZP_PET	0.000
**40**	SZHGE_PET	0.005
**41**	LZLGE_PET	0.001
**42**	LZHGE_PET	0.001
**43**	GLV_PET.1	0.000
**44**	Contrast_PET.1	0.000
**45**	Complexity_PET	0.209
**46**	Coarseness_PET	0.000
**47**	Variance_PET.2	0.001

Table S3 **Univariate Analysis (*continuation*)**		
**No.**	**Variable**	***p*-value (2-side)**
**48**	CodeEntropy_PET	0.027
**49**	Contrast_PET.2	0.000
**50**	IDM_PET	0.106
**51**	Entropy_PET.3	0.027
**52**	BWS_PET	0.000
**53**	MaxSpe_PET	0.043
**54**	Skewness_CT	0.193
**55**	Kurtosis_CT	0.243
**56**	Entropy_CT.1	0.191
**57**	Correlation_CT	0.156
**58**	Clusterprominence_CT	0.084
**59**	Clustershade_CT	0.015
**60**	Sumentropy_CT	0.063
**61**	ICM1_CT	0.812
**62**	ICM2_CT	0.307
**63**	Variance_CT.1	0.029
**64**	C.MaxPossibility_CT	0.109
**65**	IDN_CT	0.003
**66**	GLF_CT	0.309
**67**	GaLN_CT	0.000
**68**	SGLGE_CT	0.020
**69**	SGHGE_CT	0.933
**70**	LGLGE_CT	0.299
**71**	LGHGE_CT	0.000
**72**	GrLV_CT	0.238
**73**	GaLV_CT	0.137
**74**	Energy_CT.2	0.246
**75**	GLN_CT	0.000
**76**	SRLGE_CT	0.001
**77**	RLV_CT	0.493
**78**	SZE_CT	0.004
**79**	ZSNv_CT	0.000
**80**	ZP_CT	0.005
**81**	SZLGE_CT	0.000
**82**	LZLGE_CT	0.390
**83**	LZHGE_CT	0.000
**84**	GLV_CT.1	0.147
**85**	ZSV_CT	0.000
**86**	Strength_CT	0.000
**87**	Contrast_CT.1	0.001
**88**	Busyness_CT	0.002
**89**	Complexity_CT	0.329
**90**	Variance_CT.2	0.000
**91**	CodeSimilarity_CT	0.086
**92**	Contrast_CT.2	0.008
**93**	IDM_CT	0.039
**94**	BWS_CT	0.003
**96**	MaxSpe_CT	0.115

Figure S1. Cross-validated estimation of the best alpha parameters for Lasso. The mean squared error was used as the cross-validation (CV) score, where higher values are better than lower values. Alpha was determined to be 0.0064. Lasso = least absolute shrinkage and selection operator, std = standard deviation.

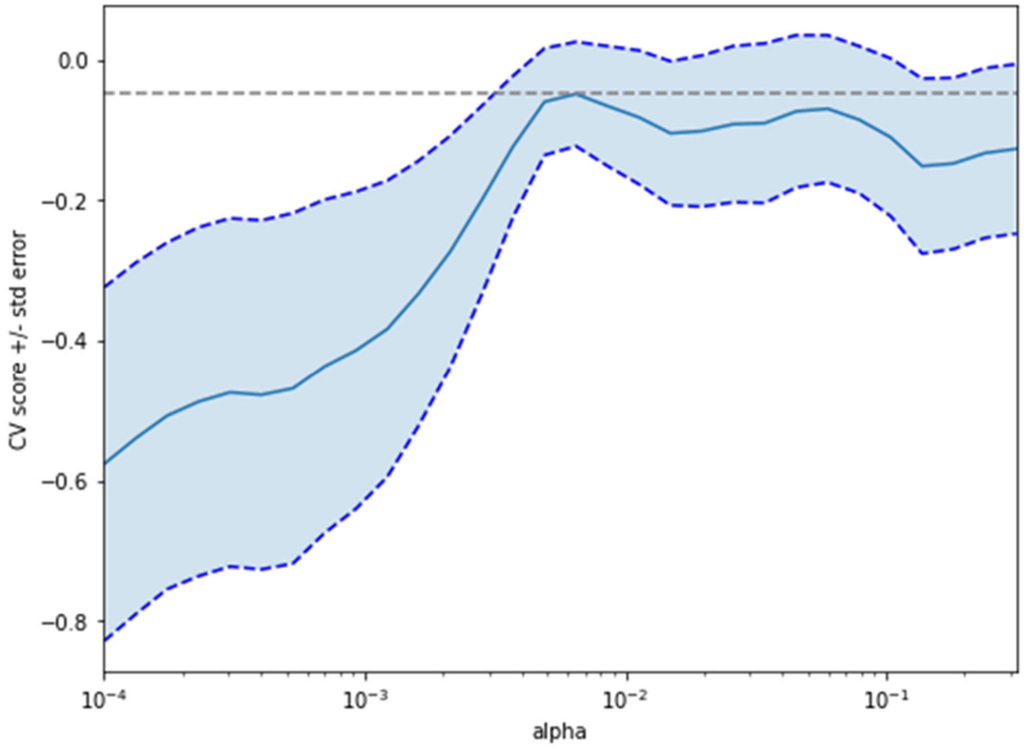

Table S4. **Selected features by Lasso (no ranked by predictive importance)**

**SVM (13)**<br>'Kurtosis_PET' 'PR' 'Δ-Grading' 'Side (R=1, L=2)' 'Δ-ER' 'Her2' 'Her2_META' 'Skewness_CT' 'PR-MET' 'BWS_PET' 'P53' 'SUV_max' 'T'<br><br>**Naive-Bayes (5)**<br>'Kurtosis_PET' 'PR' 'Δ-Grading' 'Side (R=1, L=2)' 'Δ-ER'<br><br>**Random Forest (14)**<br>'Kurtosis_PET' 'PR' 'Δ-Grading' 'Side (R=1, L=2)' 'Δ-ER' 'Her2' 'Her2_META' 'Skewness_CT' 'PR-MET' 'BWS_PET' 'P53' 'SUV_max' 'T' 'M'<br><br>**Logistic Regression (47)**<br>'Kurtosis_PET' 'PR' 'Δ-Grading' 'Side (R=1, L=2)' 'Δ-ER' 'Her2' 'Her2_META' 'Skewness_CT' 'PR-MET' 'BWS_PET' 'P53' 'SUV_max' 'T' 'M' 'Contrast_PET.2' 'Contrast_CT.2' 'Variance_CT.2' 'Variance_PET.2' 'N' 'Age at Diagnose' 'LZLGE_CT' 'Strength_CT' 'BWS_CT' 'Kurtosis_CT' 'ER-META' 'ZSV_CT' 'Variance_PET' 'ZSNv_CT' 'SGHGE_CT' 'Min_PET' 'Std_Dev_PET' 'Complexity_PET' 'GLN_CT' 'Mean_PET' 'SZHGE_PET' 'Max_PET' 'LGHGE_PET' 'Complexity_CT' 'LZHGE_PET' 'LGHGE_CT' 'Clustershade_CT' 'GLV_CT.1' 'Clusterprominence_PET' 'GLV_PET.1' 'Sum_PET' 'Clusterprominence_CT' 'LZHGE_CT'<br><br>**KNN (15)**<br>'Kurtosis_PET' 'PR' 'Δ-Grading' 'Side (R=1, L=2)' 'Δ-ER' 'Her2' 'Her2_META' 'Skewness_CT' 'PR-MET' 'BWS_PET' 'P53' 'SUV_max' 'T' 'M' 'Contrast_PET.2'<br><br>**AdaBoost (12)**<br>'Kurtosis_PET' 'PR' 'Δ-Grading' 'Side (R=1, L=2)' 'Δ-ER' 'Her2' 'Her2_META' 'Skewness_CT' 'PR-MET' 'BWS_PET' 'P53' 'SUV_max'<br><br>**Neural Network (14)**<br>['Kurtosis_PET' 'PR' 'Δ-Grading' 'Side (R=1, L=2)' 'Δ-ER' 'Her2' 'Her2_META' 'Skewness_CT' 'PR-MET' 'BWS_PET' 'P53' 'SUV_max' 'T'

Lasso = least absolute shrinkage and selection operator

Figure S2. Predictor importance for models with Lasso

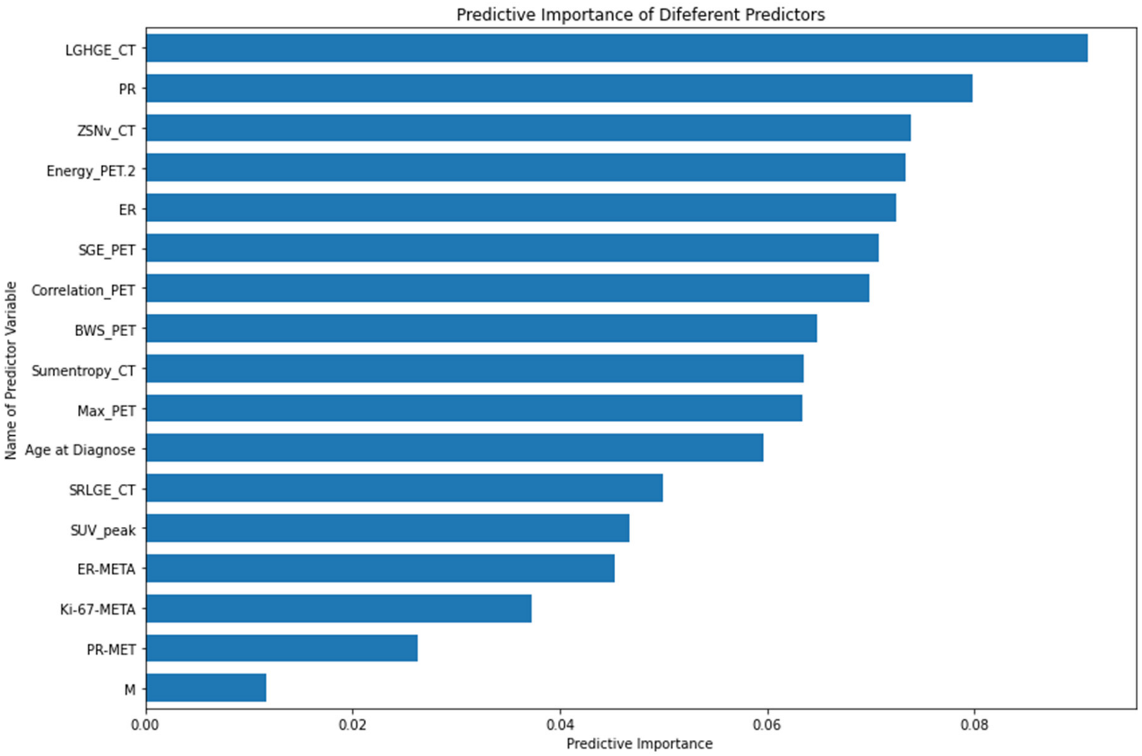

Supplement: Supplementary file 1 [file cancers-14-02922-s001.zip › cancers-1713585-supplementary.pdf]
